# Potential for Phytoremediation of PCDD/PCDF-Contaminated Sludge and Sediments Using *Cucurbitaceae* Plants: A Pilot Study

**DOI:** 10.1007/s00128-016-1868-6

**Published:** 2016-06-30

**Authors:** Magdalena Urbaniak, Anna Wyrwicka, Marek Zieliński, Joanna Mankiewicz-Boczek

**Affiliations:** 1 European Regional Centre for Ecohydrology of the Polish Academy of Sciences, Tylna 3, 90-364 Lodz, Poland; 2 Department of Applied Ecology, Faculty of Biology and Environmental Protection, University of Lodz, Banacha 12/16, 90-237 Lodz, Poland; 3 Department of Plant Physiology and Biochemistry, Faculty of Biology and Environmental Protection, University of Lodz, Banacha 12/16, 90-237 Lodz, Poland; 4 Nofer Institute of Occupational Medicine, Teresy 8, 91-348 Lodz, Poland

**Keywords:** Sewage sludge, Urban sediments, Phytotoxicity, Phytoremediation

## Abstract

The current study evaluates the impact of sewage sludge and urban reservoir sediment on changes in total and Toxic Equivalency (TEQ) PCDD/PCDF concentration in soil and phytotoxicity measured using three test species: *Lepidium sativum,Sinapis alba,* and *Sorghum saccharatum*, during 5 weeks of *Cucurbita pepo* L. cv ‘Atena Polka’ (zucchini) cultivation. ‘Atena Polka’ was found to reduce total PCDD/PCDF concentration by 37 % in soil amended with sludge and 32 % in soil treated with sediment from an urban reservoir. The TEQ reduction was almost twofold greater: 68 % in soil amended with sludge and 52 % with urban sediment. Addition of sludge increased root growth inhibition of *L. sativum,S. alba* and *S. saccharatum,* from 44 % to 90 %. Observed inhibitions were, however, reduced by ‘Atena Polka’ cultivation, and as high as 32 % promotion in root length was noted. Amendment with urban sediment, in turn, resulted in an initial 1 %–36 % promotion of root growth, while ‘Atena Polka’ cultivation reduced this positive effect by inhibition as high as 26 %. Results demonstrated positive influence of ‘Atena Polka’ on the phytotoxicity alleviation and mitigation of total and TEQ PCDD/PCDF concentrations in soil treated with bio-solids from sewage sludge and an urban reservoir.

**Electronic supplementary material:**

The online version of this article (doi:10.1007/s00128-016-1868-6) contains supplementary material, which is available to authorized users.

Production of sewage sludge is steadily increasing, and the question of its safe and responsible disposal is gaining importance. Similarly, sediments accumulated in small urban water bodies need to be periodically dredged to avoid reservoir siltation, while also utilizing them in a safe way due to impurities they contain, such as polychlorinated dibenzo-*p*-dioxins (PCDDs) and polychlorinated dibenzofurans (PCDFs), whose storage and utilization represent a risk for the environment.

Most PCDDs and PCDFs are characterized by high hydrophobicity, expressed by their high log K_ow_ value. Studies have shown substances with a log K_ow_ > 3.5 are not bioavailable to plants, since they are strongly adsorbed by soil particles and do not pass into the soil solution from which they could be taken up (Briggs et al. 1982). By comparison, toxic congeners of PCDD/PCDF are characterized by log K_ow_ values ranging from 6.53 to 8.78. Studies have reported vegetables (Lovett et al. 1997), fruits (Müller et al. 1993), rice (Ugeaki et al. 2006) and grasses and weeds (Reischl et al. 1989) are unable to phytoextract, transport and accumulate PCDDs/PCDFs in their tissues.

Exceptions to this are plants of the *Cucurbitaceae* family, which are able to take up PCDDs/PCDFs, as well as other POPs such as PCBs, from the soil and translocate them to stems and leaves (Wyrwicka et al. 2014; Greenwood et al. 2011; Low et al. 2011; Low and Whitfield Aslund 2010; Inui et al. 2008; Engwall and Hjelm 2000; Hülster et al. 1994). However, as considerable differences have been observed to date between the uptake and translocation of selected POPs, the correct selection of an appropriate *Cucurbitaceae* species/cultivar is an important consideration in maximizing phytoremediation efficiency (Inui et al. 2008). This is important in bio-solid amended soils, which contain a vast array of organic and inorganic compounds with varied properties and toxicity.

The aim of this study was to evaluate the suitability of the most common *Cucurbita pepo* L. cultivar in Poland—*C. pepo* L. cv ‘Atena Polka’ (zucchini)—as a phytoremediation tool for soil contaminated with PCDDs/PCDFs from sewage sludge and urban reservoir sediment. The impact of sewage sludge and urban reservoir sediment on changes in soil toxicity and phytotoxicity were measured before and after 5 weeks of *C. pepo* L. cv ‘Atena Polka’ cultivation, the former measured as total and Toxic Equivalency (TEQ) PCDD/PCDF concentration, and the latter using three test species: *Sinapis alba* L.*, Lepidium sativum* L. and *Sorghum saccharatum* (L.) Moench.

## Materials and Methods

Sewage sludge from the Lodz Municipal Wastewater Treatment Plant and urban sediments from the sedimentation pond constructed on the Sokołówka River in Lodz (Central Poland) were collected and used as soil additives (Wyrwicka et al. 2014). Sewage sludge and sediments were dried at 70°C for 72 h, homogenized into small particles using a mortar, and then used as an additive for the vegetable potting soil. Vegetable potting soil (specified for *Cucurbitaceae* growth) used in the experiment was collected from Hollas Sp. z o.o. Pasłęk.

Three study groups with 1.8, 5.4 or 10.8 g sewage sludge/urban sediment per 300 mL flower pot were used, as well as a control in which no sludge or urban sediment was added. Each treatment variant was prepared in three replicates. Doses used constituted 1.5, 4 and 8 % of the total dry weight of sample. The 1.8 g treatment corresponded to a dose of 3 tonnes per hectare (t/ha), the permitted annual dosage of municipal sewage sludge by the Ministry of the Environment Regulations (Journal of Laws 2015, item 257) while 5.4 g represented the permitted dose of 9 t/ha applied on one occasion per 3 years. The above permissible doses of sludge, despite effectively protecting the soil environment against pollution (e.g. by metals), largely limit the possibilities of enriching soil organic matter and thus increasing soil fertility. Therefore to assess the impact of high amounts of sludge and sediments on soil pollution measured as PCDDs/PCDFs and its phytotoxicity, the study also used a dose of 10.8 g, which corresponds to 18 t/ha, thus significantly exceeding the permitted level. Physico-chemical properties of used soil and soil amended with 9 t/ha of each additive are depicted in Table 1S.

*Cucurbita pepo* L. cv ‘Atena Polka’ seeds were germinated in Petri dishes for 7 days. Seedlings were planted in the control and sewage sludge- or sediment-amended soil samples and then cultivated for a 5-week period in a growth chamber at 23°C (±0.5°C) with 16 h light/8 h dark cycle and 150 mmol m^−2^ s^−1^ photon flux density during the light period, with 60 % relative humidity.

Analysis of the 17 toxic congeners of PCDD/PCDF was performed according to PN-EN 1948-2(2002) and US EPA Method 1613 (1994) using the isotope dilution method and 6890 N High Resolution Gas Chromatography/High Resolution Mass Spectrometry system (Agilent Technologies) with a DB-5MS column (Urbaniak et al. 2014). Final results were expressed as the TEQ of each sample, operationally defined by the sum of the concentrations of each congener in the mixture multiplied by its Toxic Equivalency Factor (TEF) (Van den Berg et al. 2006). All analytical work was performed in the Laboratory of Environmental Biochemistry at the Nofer Institute of Occupational Medicine, Lodz, Poland. Quantification was performed using certified calibration standards. Each analytical batch contained a sample blank, control sample, certified reference material and in-house control material. Analyte recoveries were determined by analyzing samples spiked with PCDD/PCDF standards. A reagent blank was used to assess artifacts, the precision was verified by duplicate analyses, and recoveries were estimated using samples spiked with PCDD/PCDF. Sample spikes were used to further confirm accuracy. LOD values obtained through the analytical procedure ranged from 0.068 to 0.137 ng/kg for PCDDs and 0.053 to 0.143 ng/kg for PCDFs.

The Phytotoxkit™ test kit (Microbiotests inc, Belgium) was used to assess phytotoxicity of soil before and after *C. pepo* L. cv ‘Atena Polka’ cultivation. Three test plant species were used: the monocotyledon *S. saccharatum* (L.) Moench and the dicotyledons *L. sativum* (L.) and *S. alba* (L.). Response of test species was classified as toxic when the percentage effect of root growth inhibition ≥20 % (Persoone et al. 2003).

All results were subjected to statistical analyses using Statistica software for Windows. The Wilcoxon matched-pair test was used to compare changes in total and TEQ concentrations, as well as soil sample phytotoxicity, before and after *C. pepo* L. cv ‘Atena Polka’ cultivation.

## Results and Discussion

Plants have been frequently shown to remove POPs from soils (Zhao et al. 2006; Susarla et al. 2002; Macek et al. 2000). The high propensity of selected *Cucurbitaceae* to extract PCDDs/PCDFs from soil was first reported by Hülster et al. (1994), who found that *C. pepo* L. fruits contained double the PCDD/PCDF concentrations of other examined plants. Zhang et al. (2009) reported that *C.pepo* L. roots effectively enable the uptake and subsequent translocation of PCDDs/PCDFs to aboveground parts. Inui et al. (2008) found the *C. pepo* cv ‘Patty Green’, ‘Gold Rush’ and ‘Black Beauty’ to have varied PCDD/PCDF phytoextraction capacities. Although aerial parts of ‘Patty Green’ demonstrated TEQ levels twice those found in tobacco plants, ‘Black Beauty’ and ‘Gold Rush’ demonstrated TEQ levels 180-fold higher than those of Patty Green. These studies were based mostly on the PCDD/PCDF concentrations in plant tissue (roots, leaves, fruits) and did not exhibit their changes in soil due to *C. pepo* L. cultivation. Moreover, available literature data concerning removal of PCDDs/PCDFs by *C. pepo* L. monitored mostly changes of single congener or group of congeners over time (Zhang et al. 2009; Campanella and Paul 2000; Hülster and Marschner 1994). The present study assessed the efficacy of *C. pepo* L. as a phytoremediation tool for soil contaminated due to application of bio-solids – a mixture of different compounds with varied properties and toxicity.

**Fig. 1:**
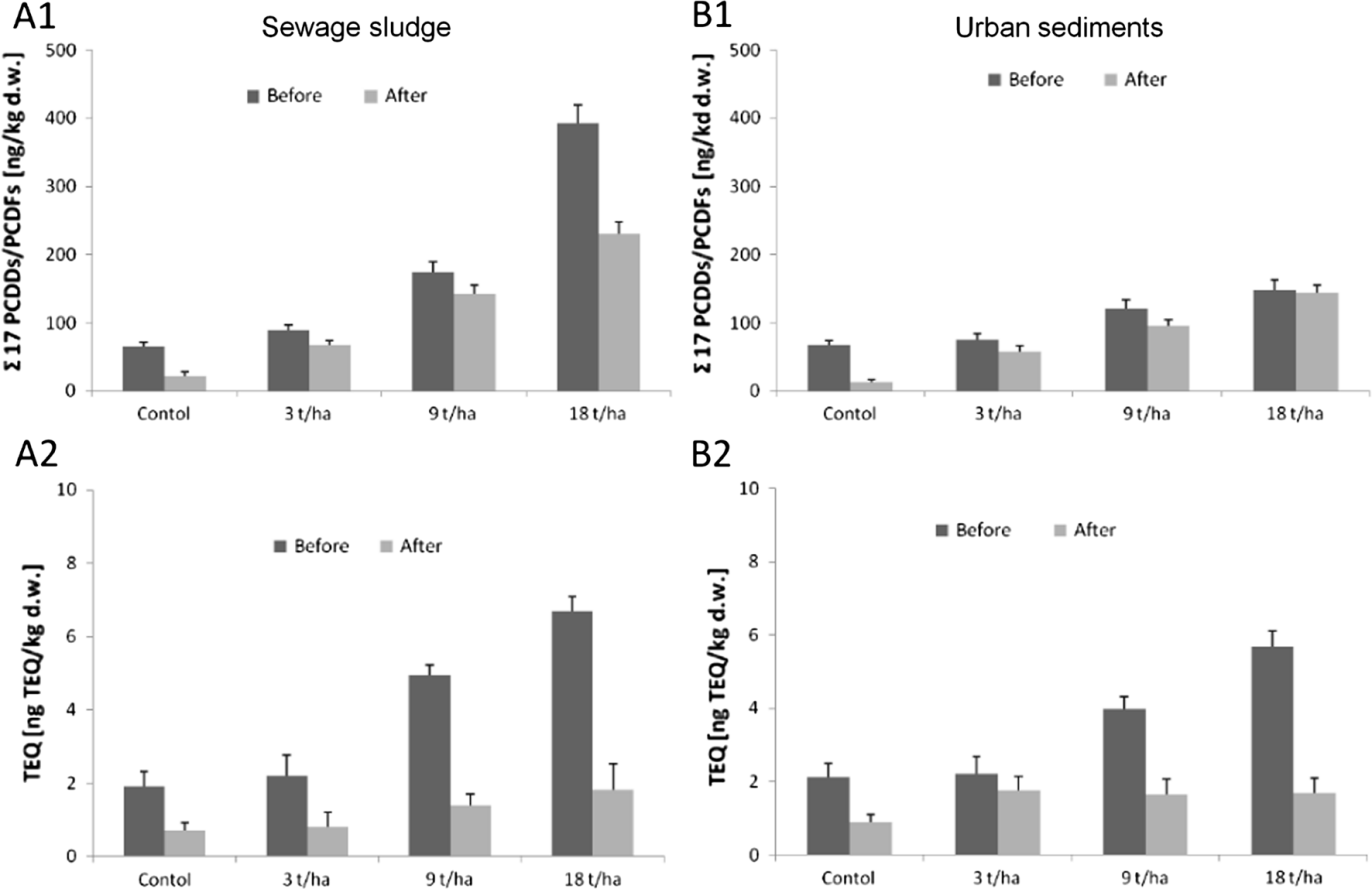
Mean decreases in total and TEQ PCDD/PCDF concentrations in soil amended with different doses of sewage sludge (**A1**, **A2**) and urban sediments (**B1**–**B2**) before and after *Cucurbita* *pepo* L. cv ‘Atena Polka’ cultivation

Current results demonstrated that cultivation of *C. pepo* L. cv. ‘Atena Polka’ reduced total PCDD/PCDF content by a mean value of 37 % in soil amended with sewage sludge and 32 % in soil treated with urban sediment (Fig. 1A1, B1; Table 2S). Mean reduction in TEQ concentrations were 68 and 52 % in soil amended with sewage sludge and sediment, respectively; values almost twice those of PCDD/PCDF content (Fig. 1A2, B2; Table 2S). Wilcoxon matched pair test revealed significant differences in total and TEQ values before and after *C. pepo* L. cv. ‘Atena Polka’ cultivation at *p* = 0.067. The greatest decline of total PCDD/PCDF content was observed for control samples (66 % for soil with sewage sludge and 81 % for soil with sediment), while the greatest reduction of TEQ values was detected in samples fertilized with 9 and 18 t/ha of sewage sludge (72 and 73 %, respectively) (Fig. 1; Table 2S). In soil amended with 3 t/ha of sludge, ‘Atena Polka’ cultivation led to a 63 % reduction of TEQ. Other large decreases were also noted for soil amended with 9 and 18 t/ha of urban sediments (59 and 70 %, respectively), while a much smaller reduction (21 %) was noted for a dose of 3 t/ha (Fig. 1; Table 2S). The above declines in soil total and TEQ PCDD/PCDF concentrations are, from one site, a result of ‘Atena Polka’ cultivation, however, the bioremediation activity of soil microorganisms seems to also be an important factor responsible for the obtained reductions (Urbaniak 2013; Field and Sierra-Alvarez 2008).

The study also showed that, while observed declines in TEQ reduction were found to be irrespective of sewage sludge concentration (63, 72 and 73 % for 3, 9 and 18 t/ha, respectively) (Fig. 1A2; Table 2S), for samples amended with urban sediments, removal efficiency significantly increased (*p* = 0.067) together with sediment dose (21, 59 and 70 % for 3, 9 and 18 t/ha, respectively) (Fig. 1B2; Table 2S). Greater differences in reduction rates were observed between samples treated with lower doses of sludge and sediment: 42, 13 and 3 % differences for 3, 9 and 18 t/ha, respectively (Fig. 1A2, B2; Table 2S). Similarly Wyrwicka et al. (2014) reported that the use of *Cucumis sativus* L. led to a greater reduction of PCBs in samples treated with sewage sludge than those treated with urban sediment, and similar increases in PCB reduction were found with increasing sediment treatment.

**Fig. 2:**
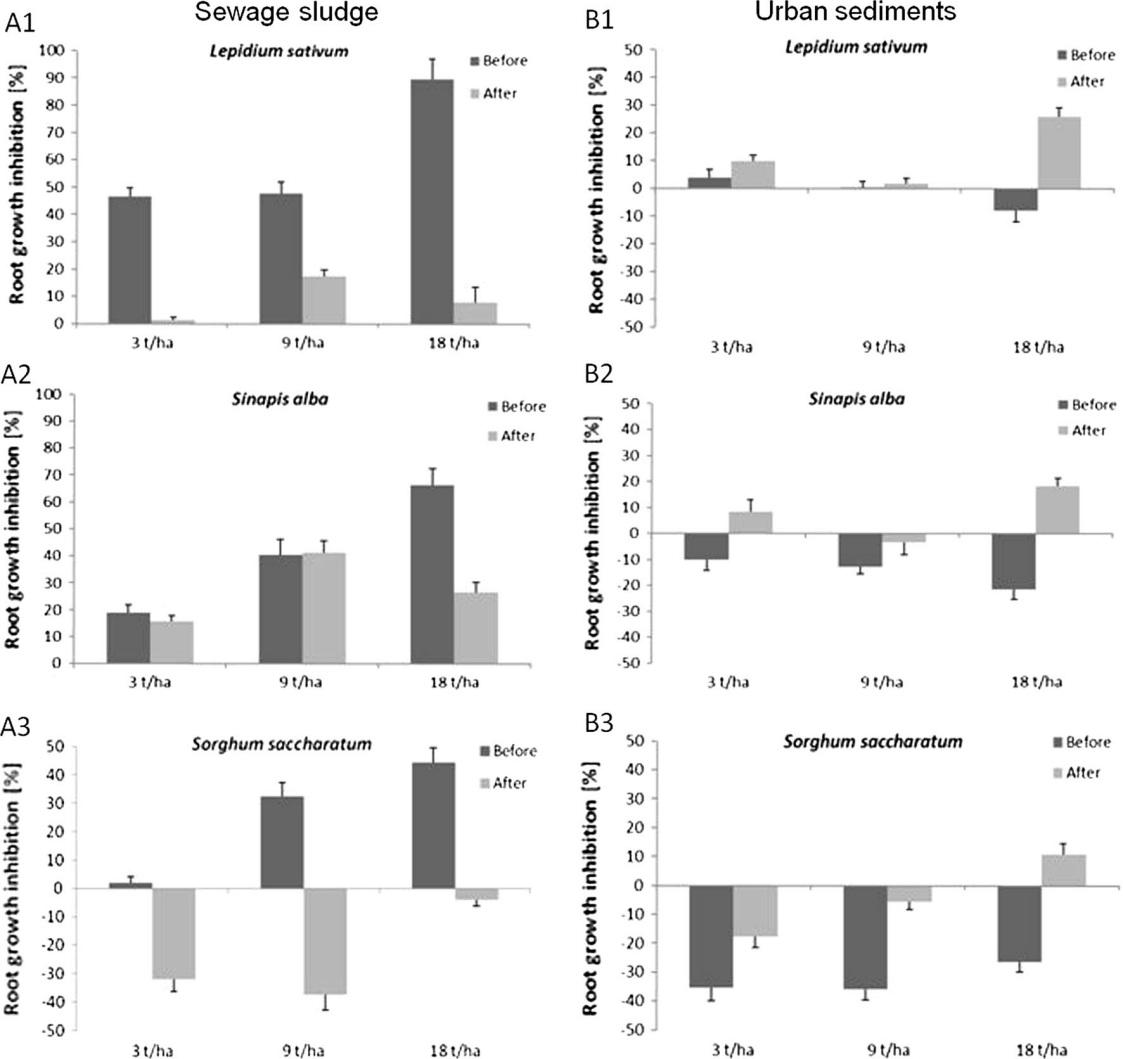
Mean effect of different doses of sewage sludge (**A1**–**A3**) and urban sediments (**B1**–**B**3) on root growth inhibition before and after *Cucurbita* *pepo* L. cv ‘Atena Polka’ cultivation (negative values indicated increase in the root growth in comparison to the control soil)

‘Atena Polka’ was also found to play a positive role on changes in soil phytotoxicity. Although high root growth inhibition was initially observed for *S. saccharatum* (44 %)*, S. alba* (66 %) and *L. sativum* (90 %) in soil treated with 18 t/ha sewage sludge (Fig. 2A1–A3), these values decreased after ‘Atena Polka’ cultivation: 26 % in the case of *S. alba* (Fig. 2A2), 8 % for *L. sativum* and 4 % for *S. saccharatum*. A similar situation was observed for lower doses of sludge. The greatest inhibition of root growth was observed for *L. sativum*: 46 % (3 t/ha) and 47 % (9 t/ha) before ‘Atena Polka’ cultivation, falling to 1 % (3 t/ha) and 17 % (9 t/ha) after 5 weeks of plant cultivation (Fig. 2A1). Much greater alleviation of soil toxicity was demonstrated by *S. saccharatum,* with a 32 % increase in root growth observed at 3 t/ha and 27 % for 9 t/ha (Fig. 2A3), while *S. alba* demonstrated the lowest reduction in soil toxicity (Fig. 2A2). No significant differences in soil phytotoxicity before and after ‘Atena Polka’ cultivation (*p* = 0.109) were noted.

Opposite results were observed for samples amended with urban sediments. No toxic effects were observed, as all test species showed an increase in root growth, with the greatest being observed for *S. sacharatum* (36 %) (Fig. 2B1–B3). However, this positive effect diminished after application of ‘Atena Polka’, with root growth inhibition as high as 26 % (Fig. 2B1–B3).

Results indicate that while applied sewage sludge negatively affected growth of all test plants, the degree of inhibition depended on the plant type. The greatest tolerance to sewage sludge was shown by *S. saccharatum* and the lowest by *L. sativum*. Similar results, with the high resistance of *S. saccharatum* and sensitivity of *L. sativum* to sludge application, were obtained in the study by Oleszczuk and Hollert (2011). High sensitivity of *L. sativum* has also been confirmed in other studies worldwide (Oleszczuk et al. 2012; Ramirez et al. 2008; Alvarenga et al. 2007). Such high phytotoxicity could possibly be due to the composition of the amendment, as sewage sludge contains a range of pollutants other than PCDDs/PCDFs, such as PCBs (Wyrwicka et al. 2014) and metals, which usually exceed the allowed doses (Journal of Laws 2015, item 257) and can negatively affect the condition of the soil and the plant. Urban sediments, in turn, are rich in phosphorus, iron and calcium which are valuable for arable farming and the reconditioning of sandy and degraded soils, while metals remain low (see Table 1S). Urban sediments alleviate toxic effects of PCDDs/PCDFs and other pollutants, which manifest themselves in increased *L. sativum, S. alba* and *S. saccharatum* root growth. Further decreases observed in the positive effects of sediment treatment associated with ‘Atena Polka’ cultivation can be related to soil impoverishment.

With regard to the mitigation of soil phytotoxicity as an effect of *C. pepo* cultivation, the highest average reduction was demonstrated by *S. saccharatum* (59 %) and *L. sativum* (52 %), while *S. alba* showed the lowest average decline (14 %). Obtained differences may be related to the sensitivity of test plants to soil pollution. While *C. pepo* is effective for the removal of organic compounds, it is not sufficient to remove metals. The high inhibition of *S. alba* roots observed following *C. pepo* cultivation may be related to the mix of pollutants remaining in soil after the phytoremediation process. Baran and Tarnawski (2015) reported *S. alba* to be particularly sensitive to metals, while Steliga et al. (2012) noted that it is not susceptible to soil organic contaminants.

Results demonstrate that cultivation of ‘Atena Polka’ plays a positive role in reducing total PCDD/PCDF soil concentration and TEQ equivalent, with the highest reduction efficiency observed in soil treated with sewage sludge. Administration of sludge was found to result also in high inhibition of root growth by the test plants *L. sativum, S. alba* and *S. saccharatum*, while the application of ‘Atena Polka’ alleviated the negative effects of sludge usage. Opposite results were obtained for soil amended with urban sediments, which demonstrated not only lower total PCDD/PCDF concentrations and hence, lower efficiency in their removal, but also a lack of toxic response of *L. sativum, S. alba* and *S. saccharatum*. Results demonstrated the positive influence of ‘Atena Polka’ on phytotoxicity alleviation and mitigation risk related to PCDDs/PCDFs in soil treated with bio-solids. Results confirmed potential of the used cultivar in remediation of soils contaminated with bio-solids containing a mixture of different compounds with varied properties and toxicity.

## Electronic supplementary material

Below is the link to the electronic supplementary material.

Supplementary material 1 (DOCX 20 kb)
