# Supplementary material for: Potential for Phytoremediation of PCDD/PCDF-Contaminated Sludge and Sediments Using Cucurbitaceae Plants: A Pilot Study
Source: Bull Environ Contam Toxicol. 2016 Jun 30;97:401–6. doi: 10.1007/s00128-016-1868-6 (PMC4978765; doi:10.1007/s00128-016-1868-6)
Supplement: Supplementary file 1 — Supplementary material 1 (DOCX 20 kb) [file 128_2016_1868_MOESM1_ESM.docx]

**Supplementary materials**

**Table 1S.** Physico-chemical properties of the used soil. and soil amended with sewage sludge and sediments in dose of 9 t/ha

| **Properties** | **Control soil** | **Soil mixed with 9 t/ha of sewage sludge** | **Soil mixed with 9 t/ha of sediments** |
| --- | --- | --- | --- |
| pH | 6.0+/-0.4 | 6.2+/-0.4 | 6.2+/-0.4 |
| Total Organic Carbon [g/kg] | 15.18 | 22.67 | 13.89 |
| Total Nitrogen  [%] | 0.52+/-0.08 | 0.66+/-0.10 | 0.50+/-0.07 |
| N-NO_3_  [mg/kg d.w.] | 782+/-234 | 859+/-257 | 756+/-226 |
| P  [mg/kg d.w.] | 437+/-88 | 728.7+/-146 | 446.23+/-89.89 |
| K  [mg/kg d.w.] | 2341+/-445 | 2809+/-534 | 2586+/-491 |
| Ca  [mg/kg d.w.] | 2640+/-501 | 2755+/-523 | 2915+/-554 |
| Mg  [mg/kg d.w.] | 1099 | 1221 | 1040 |
| Cl  [mg/kg d.w.] | 69.4+/-17.4 | 127.3+/-31.8 | 79.77+/-19.90 |
| Na  [mg/kg d.w.] | 150 | 190 | 144 |
| Pb  [mg/kg d.w.] | 5.7+/-1.4 | 7.1+/-1.8 | 6.7+/-1.7 |
| Cd  [mg/kg d.w.] | <0.21 | <0.21 | <0.21 |
| Cr  [mg/kg d.w.] | <4.2 | 31.3+/-6.4 | <4.2 |
| Cu  [mg/kg d.w.] | 22.2+/-4.6 | 31.3+/-6.4 | 24.2+/-5.0 |
| Ni  [mg/kg d.w.] | 4.9+/-1.1 | 6.4+/-1.5 | 5.8+/-1.3 |
| Zn  [mg/kg d.w.] | 22.4+/-4.2 | 72.8+/-13.6 | 31.8+/-5.9 |
| Fe  [mg/kg d.w.] | 2297+/-430 | 3311+/-619 | 2750+/-514 |
| Mn  [mg/kg d.w.] | 115+/-23.7 | 112.3+/-23.1 | 118.4+/-24.4 |

**Materials and Methods**

**PCDD/PCDF analysis**

The pretreatment of the sediment samples were performed according to PN-EN 1948-3 (2002) and EPA Method 1613. Two grams of each sample was spiked with isotopically labeled standards (Cambridge Isotopes Laboratories. USA) and extracted by Accelerated Solvent Extraction 200 Dionex at 150 atm (11 MPa). and the oven was heated to 175 °C with toluene. The extracts were purified with multilayer silica columns packed with neutral. acidic. and basic silica gel and eluted with 200 mL of hexane. The hexane extracts were further concentrated to 5.00 mL by rotary evaporation and concentrated to 100 μL under a gentle stream of nitrogen. replacing the n-hexane with nonane.

The identification and quantification of seven congeners of PCDDs and 10 congeners of PCDFs. identified by the WHO as potentially toxic were performed by high-resolution gas chromatography (HRGC)/high-resolution mass spectrometry (HRMS): an HP 6890 N Agilent Technologies GC (Santa Clara. USA) coupled with a high resolution mass spectrometer (AutoSpec Ultima) (Milford. USA). The GC was operated in the splitless injection mode and. for the HRMS. perfluorokerosene was used as a calibration reference (lock mass).

The oven temperature protocol was 150 °C for 2 min. 20 °C min^−1^ to 200 °C (0 min). 1 °C min^−1^ to 220 °C for 16 min and 3 °C min^−1^ to 320 °C for 3 min. The injector temperature was 270 °C. The mass spectrometer was operated under positive electron ionization conditions: 34.8 eVelectron energy at a resolving power of 10.000 with an ion source temperature of 250 °C. Helium was used as a carrier gas at a flow rate of 1.60 mL min^−1^. Samples were quantified with the isotope dilution method.

**Results and discussion**

**Table 2S.** Changes in total and TEQ PCDDs/PCDF concentrations in soil amended with different doses of sewage sludge and urban sediment before and after *Cucurbita pepo* L. cv. ‘Atena Polka’ cultivation

| Compounds | **Sewage sludge** | | | | | | | | |
| --- | --- | --- | --- | --- | --- | --- | --- | --- | --- |
|  | Before *Cucurbita pepo* L. cv ’Atena Polka’ cultivation | | | |  | After *Cucurbita pepo* L. cv ’Atena Polka’ cultivation | | | |
|  | Control | 3 t/ha | 9 t/ha | 18 t/ha |  | Control | 3 t/ha | 9 t/ha | 18 t/ha |
| Total PCDD/PCDF  [ng/kg d.w.] | 64.83 | 89.20 | 174.55 | 392.67 |  | 22.29 | 67.83 | 142.79 | 230.50 |
| TEQ  [ng TEQ/kg d.w.] | 1.91 | 2.20 | 4.94 | 6.69 |  | 0.71 | 0.81 | 1.40 | 1.82 |
|  | **Urban sediments** | | | | | | | | |
| Total PCDD/PCDF  [ng/kg d.w.] | 67.60 | 74.45 | 121.30 | 147.00 |  | 12.82 | 57.12 | 94.83 | 144.34 |
| TEQ  [ng TEQ/kg d.w.] | 2.12 | 2.21 | 3.99 | 5.69 |  | 0.89 | 1.75 | 1.63 | 1.70 |
